# Evaluating the Effectiveness of a Family-Based Lifestyle Intervention for Managing Childhood Overweight: Protocol for a Randomized Controlled Trial

**DOI:** 10.2196/76837

**Published:** 2025-10-14

**Authors:** R Rebecca Jantzen, Patti-Jean Naylor, Karen Strange, Geoff D C Ball, Louise C Mâsse, Ryan E Rhodes, Xuekui Zhang, Robert P Nolan, Sarah Zheng, Valeria Rac, Sam Liu

**Affiliations:** 1 School of Exercise Science, Physical & Health Education University of Victoria Victoria, BC Canada; 2 Childhood Healthy Living Foundation Victoria, BC Canada; 3 Pediatrics Department Faculty of Medicine & Dentistry University of Alberta Edmonton, AB Canada; 4 School of Population and Public Health Faculty of Medicine University of British Columbia Vancouver Canada; 5 BC Children's Hospital Research Institute Vancouver, BC Canada; 6 Department of Mathematics & Statistics University of Victoria Victoria, BC Canada; 7 Behavioural Cardiology Research Unit, Department of Medicine Peter Munk Cardiac Centre University Health Network Toronto, ON Canada; 8 Gustavson School of Business University of Victoria Victoria, BC Canada; 9 Institute of Health Policy, Management and Evaluation Dalla Lana School of Public Health University of Toronto Toronto, ON Canada; 10 Program for Health System and Technology Evaluation, Ted Rogers Centre for Heart Research Peter Munk Cardiac Centre Toronto General Hospital Research Institute, University Health Network Toronto, ON Canada; 11 Diabetes Action Canada CIHR SPOR Network Toronto, ON Canada

**Keywords:** childhood obesity, web-based intervention, family-based intervention, health behavior change, obesity and overweight, physical activity, child behavior, parental support of child behavior

## Abstract

**Background:**

Family-focused interventions promoting lifestyle behaviors such as physical activity and healthy eating can be effective strategies for obesity management in children. To maximize public health impact, there is an urgent need to develop innovative solutions to improve the scalability of childhood obesity management interventions. Stand-alone web-based interventions are easily administered but may be hindered by low participant engagement and limited support. A blended intervention that includes both facilitated web-based group sessions and self-guided resources may enhance engagement, thereby optimizing improvements in children’s health outcomes. However, the long-term effectiveness of stand-alone versus blended web-based interventions to manage childhood obesity has not yet been evaluated.

**Objective:**

The primary objective of this randomized controlled trial is to evaluate the long-term effectiveness of a blended, web-based, family-focused healthy living program (Family Healthy Living Program [FHLP]) compared to an active control (self-guided web-based educational resources) in lowering BMI *z* scores in children with overweight or obesity over 12 months. The secondary objective is to examine intervention effects on children’s physical activity, dietary behaviors, and health-related quality of life as well as parental self-efficacy and motivation to support children’s lifestyle behaviors.

**Methods:**

This single-blind 2×4 trial aims to recruit 278 parent-child dyads across Canada to be randomly assigned to the FHLP intervention group or an active control group (1:1 ratio). The 10 weeks of FHLP activities have been designed based on the multiprocess action control framework to support children and parents in learning behavioral change skills that will enable them to improve their healthy lifestyle behaviors, and the FHLP delivery has been piloted previously. Families in the intervention group will receive weekly web-based synchronous group sessions and access to additional resources and suggested activities. Families in the control group will receive access to 10 weeks of self-guided web-based educational resources only. Outcomes, including child’s height and weight, daily physical activity, dietary behaviors, health-related quality of life, and parental support, will be measured via questionnaire at 4 assessment time points over 1 year (ie, baseline, 10 wk, 6 mo, and 12 mo). Intervention engagement and attrition will also be recorded. Multiple linear regression models will be used to examine the effectiveness of the FHLP intervention compared to stand-alone web resources only.

**Results:**

This study was funded in 2020, with 270 participants enrolled between January 2021 and May 2024. The 12-month data collection period ended in June 2025 and data analysis is currently underway. Study results are expected to be published in winter 2025. Results will be disseminated via conference presentations, peer-reviewed publications, and other media channels.

**Conclusions:**

The blended web-based FHLP intervention has the potential to become a scalable solution for the prevention of obesity and related health conditions.

**Trial Registration:**

ClinicalTrials.gov NCT06777888; https://www.clinicaltrials.gov/study/NCT06777888

**International Registered Report Identifier (IRRID):**

DERR1-10.2196/76837

## Introduction

### Background and Rationale

Childhood obesity is a prevalent pediatric health issue linked to a range of physical and psychological problems, including type 2 diabetes, hypertension, sleep apnea, and low self-esteem, all of which contribute to a diminished quality of life during childhood [1-3]. In Canada, more than 30% of children are classified as overweight, defined based on BMI ≥85th percentile for their age and sex [1]. Without effective interventions, these children have a high risk of remaining overweight into adolescence and adulthood [4]. As a result, fostering healthy eating habits and encouraging physical activity during childhood has become a central focus for policymakers and public health initiatives at the global, national, and provincial levels [5-7].

Family-focused behavioral obesity management interventions that include both parents and children as corecipients have been shown to be an effective approach for promoting healthy lifestyles and achieving weight control in children [8,9], particularly when the intervention content is grounded in behavioral theory [10]. Several randomized controlled trials have demonstrated that family-focused behavioral interventions delivered in person can be effective strategies in improving children’s lifestyle behaviors and reducing BMI *z* scores [11-13]. However, to enhance their public health impact, such interventions must be delivered through scalable and accessible methods.

Leveraging the internet to deliver these interventions can be an innovative strategy to enhance program accessibility and scalability, making it easier for families to participate regardless of their geographic location [14]. Previous studies have shown that internet-based programs can be effective in improving lifestyle behaviors and clinical outcomes, including BMI [15,16]. However, a limitation often observed in self-guided, asynchronous web-based interventions (with no human support) is reduced engagement over time [17]. By contrast, synchronous web-based sessions (delivered using video chat) may provide distinct advantages, such as fostering engagement through real-time interaction, personalized support, and opportunities for families to connect and share experiences. These sessions retain the scalability and flexibility of internet-based interventions while offering live interaction, which may enhance motivation, promote accountability, and improve adherence to the program [18]. This could make synchronous delivery a powerful and adaptable option for managing childhood obesity effectively.

Drawing on the evidence supporting the effectiveness of family-based interventions and use of the internet for program delivery, the research team partnered with stakeholders (the Childhood Healthy Living Foundation and the British Columbia Ministry of Health) to develop a 10-week early intervention lifestyle management program (Family Healthy Living Program [FHLP]) that combines web-based resources with weekly live, interactive group sessions led by trained facilitators. This intervention targets families with children aged 8 to 12 years who are above the healthy weight trajectory (BMI ≥85th percentile for age and sex). Interventions aimed at this age group are particularly impactful, as prepubertal children have a greater potential to return to a healthy growth pattern [19,20]. Extensive stakeholder consultation was conducted with more than 100 health care professionals to develop an evidence-based 10-week family-focused intervention involving both parent- and child-specific interactive activities. The FHLP intervention was designed to align with existing best practices in the clinical and public health setting, complement an existing childhood obesity management clinical intervention in British Columbia, and meet the needs of diverse families and communities [21]. The FHLP aims to support children who are off the healthy weight trajectory by managing overweight and preventing progression to obesity through targeting 5 behavioral domains: physical activity, healthy eating, sleep, media use, and mental well-being.

The FHLP was designed using the multiprocess action control (M-PAC) framework, a meta-theoretical behavior change framework emphasizing a social cognition approach to intention formation (reflective processes), the adoption of action control through self-regulation (regulatory processes), and the action control maintenance phase once a behavior becomes habitual and self-identified (reflexive processes) [22,23]. One advantage of the M-PAC framework is its ability to address the intention-behavior gap, which poses a particular challenge for childhood obesity interventions because almost all families enter these programs already intending to adopt a healthy lifestyle but often struggle to translate this intention into sustained behavior change [22,24,25]. Addressing this intention-behavior gap can therefore improve the effectiveness of physical activity interventions [23,26]. Thus, the FHLP curriculum begins with intention formation (physical and mental health benefits of physical activity as well as perceived capability), then moves into action control adoption (restructuring the physical and social environment to create opportunities for physical activity, goals and planning, and feedback and monitoring), and concludes with action control maintenance (habit formation and identity formation).

The FHLP intervention was initially piloted in 2018 [21] and delivered in person at community sites across British Columbia. The content has since been adapted for delivery as a blended web-based intervention, and previous evaluation showed a positive impact on children’s lifestyle behaviors and parental support in the short term [27,28]. However, the FHLP intervention has yet to be evaluated in a randomized controlled trial, and the long-term effectiveness of the intervention and its impact on children’s anthropometry have not been examined.

### Objectives

The primary objective of this study is to evaluate the effectiveness of the synchronous web-based FHLP intervention compared to an active control group in lowering BMI *z* scores in children (aged 8-12 y) with BMI ≥85th percentile for age and sex over a 12-month period. The secondary objectives include evaluation of the effectiveness of FHLP compared to the active control in improving lifestyle behaviors and health-related quality of life in children (aged 8-12 y) with BMI ≥85th percentile for age and sex, as well as parental self-efficacy and motivation to support their child’s healthy living behaviors. The trial hypotheses are that the FHLP intervention will result in significantly greater reduction in BMI *z* scores than the active control and that intervention participants will show greater improvements across all domains than those in the control group.

## Methods

### Trial Design

**Figure 1:**
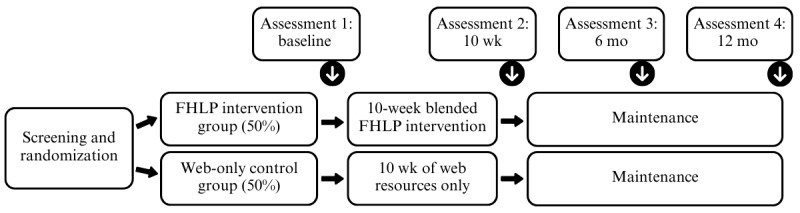
Overview of the study design and assessment timeline. FHLP: Family Healthy Living Program.

This study is a single-blind randomized controlled trial (ClinicalTrials.gov NCT06777888) with a 2×4 design (Figure 1). Participating households will be randomly assigned in a 1:1 ratio to 2 groups: FHLP intervention and control. Data will be collected at 4 assessment time points: baseline (wk 0), after the intervention (wk 10), 6-month follow-up (wk 26), and 12-month follow-up (wk 52; Figure 1). The intervention will be delivered via video chat in 3 regions across Canada (British Columbia, the prairie provinces, and Ontario), with the central site located at the University of Victoria in British Columbia.

Trial preparation, implementation, and evaluation will adhere to the CONSORT (Consolidated Standards of Reporting Trials) criteria [29], and the trial protocol will adhere to the SPIRIT (Standard Protocol Items: Recommendations for Interventional Trials) guidelines [30]. Data collection began in January 2021 and was completed in June 2025.

### Eligibility Criteria

Child participants must be aged 8 to 12 years, have BMI ≥85th percentile for age and sex, and have at least 1 primary caregiver (parent or guardian) who agrees to participate in the study along with the child. Exclusion criteria will include the inability to communicate (speak, read, and write) in English and any conditions that prevent children from being physically active.

### Recruitment

Participants will be recruited from 3 regions across Canada (British Columbia, the prairie provinces, and Ontario) using advertising via social media; local community newsletters; posters and rack cards within communities; and direct mailings to physician offices, health clinics, and allied health professionals. Interested parent-child dyads will be screened for eligibility by a trained research assistant at the central site (University of Victoria in British Columbia).

During the consent process, participants will be made aware of the differences between the 2 groups and informed that they will be randomly assigned to either the full intervention or the web resources only. Participants from both groups will be offered technical support from the research coordinator if needed for viewing web-based content, using the Fitbit Inspire tracker, or accessing the web-based questionnaires.

### Assignment of Interventions and Blinding

Participating households will be randomly assigned to the FHLP intervention or the active control group. To reduce selection bias, the centralized randomization process will be managed by a research coordinator not directly involved with intervention delivery. The research coordinator will use computer-generated random numbers to assign the participants to either the intervention or control group. Participants will not be stratified because the population is assumed to be inherently homogeneous with respect to location due to the web-based nature of the program delivery. After randomization, data will be collected electronically via REDCap (Research Electronic Data Capture; Vanderbilt University) automated invitations to reduce response bias. Other research team members, including the principal investigator, coinvestigators, and data analysts, will be blinded to group assignment to minimize detection bias and ensure impartiality in decisions regarding trial activities.

### Participant Timeline and Data Collection

The intervention period will be 10 weeks; the full assessment duration will be 12 months. Web-based questionnaire assessments will be completed at baseline and at 10-week, 6-month, and 12-month intervals after randomization (Figure 1). The assessments will include self-reported height and weight measurements and 7 days of step counts for each child as measured by Fitbit Inspire activity trackers. Questionnaire data will be collected and managed using SurveyMonkey and REDCap tools hosted at the University of Victoria. REDCap is a secure, web-based software platform designed to support data capture for research studies [31,32]. Survey responses will be stored on a secure network drive at the University of Victoria. Participants may choose to discontinue their participation at any point; data completion will be encouraged by multiple personalized email reminders and compensation at each assessment time point.

### Sample Size

The parameters used for sample size calculation are based on the results of a published randomized controlled trial evaluating the effectiveness of a family-based intervention in reducing BMI *z* scores compared to control [33]. The required sample size was estimated at 232 children, using a 2-arm, parallel-group design with 80% power. On the basis of previous research [17,33], the attrition (loss at follow-up) is expected to be similar between the groups (20%). In sum, the trial aimed to recruit a study population of 278 participants, which accounts for an attrition rate of 20%. In practice, 270 participants were successfully enrolled.

### Intervention

The FHLP intervention aims to target children who are off the healthy weight trajectory, with the goal of managing overweight and preventing progression to obesity. FHLP curriculum activities have been designed to support children and parents in learning behavioral change skills that will enable them to improve their health-related lifestyle behaviors. The details of the intervention content have been previously published [21]. Families attend weekly 2-hour web-based healthy living group sessions comprising family-based and parent-only components. Family-based components consist of interactive lessons and active games to keep children engaged in learning and enhance the reflexive processes that lead to habit formation. For the final 30 minutes of each session, parent-only components focus on food- and physical activity–related practices and strategies for improving the home environment.

The FHLP intervention will be delivered by a team of 2 facilitators with backgrounds in nutrition or physical education. These individuals will receive training from the centralized research team (based at the University of Victoria), including attending FHLP sessions delivered in British Columbia via video chat by the Childhood Healthy Living Foundation. The facilitators will be participants’ main point of contact during the 10-week intervention and will call each enrolled family (via telephone or video chat) individually before the first group session to orient them to the program. They will send weekly email reminders for group sessions and reach out to participants following absences.

During the 10-week intervention, the facilitators will deliver 1 individual orientation call and 9 weekly group sessions over Zoom (Zoom Video Communications, Inc). Weekly group sessions will follow the FHLP curriculum with modules on healthy eating, physical activity, behavior change skills, mental health, parenting practices, sleep routines, and media use. Participants will be provided with a physical kit containing hard-copy binders of the session handouts along with materials to use during session activities (eg, jump ropes, water bottles, juggling scarves, and hacky sacks). Throughout all sessions, the facilitators will accentuate behavioral change skills (cognitive and behavioral regulation processes) such as goal setting and monitoring. One group session will include a “healthy eating Q&A” led by a registered dietitian in addition to the facilitators; participants will also have the option to attend up to 3 web-based live cooking classes led by a registered dietitian and child assistant.

In addition to the facilitated weekly sessions and cooking classes, participants in the intervention group will have access to a self-guided web-based family portal offering additional resources and information, healthy recipes, articles for parents, videos, and suggested healthy eating and physical activities. The portal will also serve as a repository of materials covered in each session, maximizing flexibility in terms of participants interacting with intervention content.

### Active Control Group

It is an ethical imperative for long-term trials of health promotion interventions to ensure that the control group also receives essential information, which in this case would be education about childhood obesity management. To match the weekly intervention delivery frequency, the active control group will be given access to a similar secure portal where they can view weekly educational content aligned with the FHLP topics. This information is based on the FHLP curriculum and relevant information and resources from Canadian public health agencies (eg, Canada’s Guide to Healthy Living, Canada’s Food Guide, and Canada’s Physical Activity Guide). These materials are guideline based, user-friendly, and visually appealing; in addition, they include child-specific content.

At baseline, a member of the research team will meet with each control participant for a 10- to 15-minute video meeting to orient parents to the portal and ensure that they have sufficient internet literacy to access the resources. However, the control group will not receive other personal contact, such as the FHLP facilitated group sessions, physical kits, web-based cooking classes and Q&A sessions, regular check-ins, or email reminders to access the resources. All families in both study groups will maintain the same delivery schedule (10 weeks in total; Figure 1).

### Primary Outcome

Each child’s BMI *z* score will be calculated based on the World Health Organization criteria [34]. Height and weight will be self-reported at each time point via questionnaire, with parents provided with instructions to accurately measure their child’s height and weight at home [35].

### Secondary Outcomes

#### Child Physical Activity Behaviors

Changes in physical activity will be measured using accelerometry via Fitbit Inspire wrist-worn activity trackers. Seven consecutive days of daily step counts will be measured at each assessment time point. Changes in physical activity predilection and adequacy as well as perceived intrinsic motivation and competence in physical activity will be measured using the motivation and confidence subscale of the Canadian Assessment of Physical Literacy-2 [36]. Physical activity self-report will be measured using the Physical Activity Questionnaire for Older Children [37] and the Canadian Assessment of Physical Literacy-2 [36]. Changes in physical activity confidence, sedentary behavior, and family support for physical activity will be assessed using the Patient-Centered Assessment and Counseling for Exercise Plus Nutrition adolescent psychosocial measures [38]. Physical activity and screen time self-efficacy will be measured using the Family Life, Activity, Sun, Health, and Eating (FLASHE) questionnaire [39].

#### Child Dietary Behaviors

Changes in dietary behavior will be assessed using the 7-day recall questionnaire retrieved from the Behavioral Risk Factor Surveillance System survey questionnaire [40]. Healthy eating outcome expectations will be assessed using the Power Play! survey [41]. Dietary behavior self-efficacy will be assessed using the Patient-Centered Assessment and Counseling for Exercise Plus Nutrition adolescent psychosocial measures [38]. Healthy eating motivation will be assessed using the FLASHE and modified child nutrition questionnaires [39,42], and perceived cooking skills will be assessed using the Cooking with Kids questionnaire [43].

#### Child Mental Well-Being and Quality of Life

Changes in a child’s self-reported self-esteem, gratitude, and self-compassion will be measured using the self-esteem measure from Project EAT [44], the Gratitude Adjective Checklist [45], and the Self-Compassion Scale for Children [46], respectively. Children’s self-reported sleep quantity will be measured using the FLASHE questionnaire [39]. Changes in a child’s health-related quality of life will be assessed by parents (proxy report) using the Pediatric Quality of Life Inventory [47].

#### Parent Support for Healthy Family Lifestyle Environment

Parents will complete a questionnaire assessing changes in parental support, habit, role identity, and behavioral regulation in supporting their child’s physical activity and healthy eating practices using the Parent Support of Child Physical Activity and Eating Behaviors questionnaire [48], the Exercise Identity Scale [49], the Activity Support Scale for Multiple Groups [50], and the Perceived Behavioral Control Over Child Physical Activity Support Scale [51]. Changes in parental feeding practices and food preparation self-efficacy will be assessed using the FLASHE-EAT survey [52]. The structure of the home food environment and parents’ habits and dietary behaviors will be assessed using the Fruit and Vegetable At Home Survey for Parents [53]. Family eating habits and family physical activity habits will be measured using the Self-Report Habit Index [54].

#### Intervention Engagement and Attrition

For the control group, engagement with web content will be assessed based on the number of portal log-ins, the percentage of content accessed, and time spent viewing pages. For the FHLP intervention group, engagement will be assessed based on the number of Zoom-based group sessions attended as well as portal use (log-ins, the percentage of content accessed, and time spent viewing pages). Attrition will be recorded when a family withdraws from the study.

### Data Analysis

#### Overview

All analyses will be intention to treat and will include all participants with available data. Patterns of missing data in the study groups will be examined, and multiple imputations will be applied to address missing data if data are missing at random. Generalized linear mixed models will be used to compare the effectiveness of the 2 treatments in improving the primary outcome, BMI *z* score, over time. The models will include fixed effects for treatment group, time, and group×time interaction; in addition, they will adjust for baseline BMI *z* score and child’s sex. A random intercept will be included to account for within-participant correlations across repeated measures. Similar to the primary outcome analysis, generalized linear mixed models will be used to examine changes in secondary outcomes over time. Models will adjust for the baseline value of the outcome, child’s sex, and parent’s relationship to the child; in addition, a random intercept will be included to account for repeated measures.

Previous research has shown that parental involvement in child interventions can differ in ways that affect behavioral and weight-related outcomes [55]. Including this variable makes it possible to control for these potential sources of variability in the generalized linear mixed models.

In line with the sex- and gender-based analysis framework of the Canadian Institutes of Health Research [56], which encourages the thoughtful integration of sex and gender into health research, we have taken steps to reflect sex and gender considerations in our analysis plan. Specifically, we will adjust for child sex assigned at birth where biological differences may be relevant to health outcomes (eg, BMI trajectories). In the exploratory analysis, descriptive analyses will be conducted to explore whether intervention effectiveness differed between children’s gender or sex in the treatment group.

Engagement metrics will primarily be examined in subgroup and dose-response analyses. However, engagement may play a role in treatment effectiveness. Therefore, its potential inclusion as a covariate in secondary models will be explored. These exploratory analyses will help clarify the mechanisms underlying intervention impact.

#### Subgroup Analyses

Analyses will be performed to (1) describe intervention engagement and attrition to explore potential differences by parental income and education, study site, and ethnicity; (2) conduct descriptive analyses to explore whether intervention effectiveness differed between children’s gender or sex in the treatment group; and (3) evaluate the dose-response relationship in the intervention group and improvements in children’s health-related outcomes at 10 weeks, 6 months, and 12 months. Intervention dose will be defined by level of intervention engagement.

### Data Safety Monitoring

The University of Victoria Human Research Ethics Board considers this trial to be a minimal-risk behavioral intervention; therefore, a data safety monitoring committee will not be established.

### Ethical Considerations

#### Ethics Approval

This study has been approved by the BC Harmonized Ethics Platform (H20-00759).

#### Informed Consent and Assent

During the screening process, a research assistant will speak with parents and caregivers by telephone to inform potential participants about the trial. The research assistant will explain that a consent form will be provided along with the first set of research questionnaires. All participants will be provided with the research team’s contact information and will be encouraged to reach out if they have any questions or concerns after reading through the consent form.

Parent consent and child assent will be obtained electronically through the REDCap survey delivery system (refer to Multimedia Appendix 1 for the study consent form). To document ongoing consent, participants will be required to review and sign a copy of the consent form before accessing the parent and child surveys at each assessment time point (ie, baseline, 10 wk, 6 mo, and 12 mo). Participants may choose to discontinue their involvement at any time.

#### Compensation Details

Participants will receive grocery cards valued at CAD $25 (approximately US $18) for completing all questionnaires at each assessment point, for a total of CAD $100 (approximately US $72) incentive value over the study duration.

#### Harms or Risks to the Safety of Participants Involved in the Trial

The FHLP intervention is designed as a lifestyle behavior intervention. Participants will be exposed only to activities and information that are already readily available. The University of Victoria Research Ethics Board considers this trial minimal risk because it involves potential benefits for participants, with risks of harm that do not exceed those encountered in daily life.

Potential risks include emotional discomfort for children, such as stress or embarrassment related to research participation, particularly during weight measurement. To mitigate this, the research team will normalize the measurement as a “healthy growth check.” Research team members will also complete Balanced View BC training on minimizing weight bias and using sensitive language when discussing weight and measurements associated with weight and lifestyle behaviors [57]. In addition, research team members will receive training on scope of practice and appropriate referral procedures when issues are raised that fall outside the scope of practice of the FHLP (eg, signs of disordered eating or serious physical health concerns). Participants with such concerns will be encouraged to contact clinical health professionals.

To ensure that participants can safely participate in the physical activity component of the program, each parent or guardian will complete the Canadian Society for Exercise Physiology Get Active Questionnaire at enrollment. This is an evidence-based self-administered tool that will guide each participant’s caregiver to make an informed decision about the appropriateness of increasing their child’s physical activity [58].

#### Privacy and Confidentiality

Data collected will be deidentified for program evaluation, with participant names replaced by study ID numbers. Participants’ confidentiality and the confidentiality of the data will be protected by having no participant names on any of the data during analysis. Electronic files will be stored using participants’ unique ID numbers (ie, study ID) on a secure network drive at the University of Victoria that is accessible only to the principal investigator and research staff. Participant data will be destroyed 5 years after the final study publication.

## Results

This study was funded in January 2020, and recruitment began in January 2021. A total of 270 participants were enrolled. Data collection was completed in June 2025 and analysis is currently underway. Results are expected to be published in winter 2025.

## Discussion

### Anticipated Findings

This study aims to evaluate the effectiveness of the FHLP intervention compared to an active control group in improving BMI *z* scores and related health outcomes in children aged 8 to 12 years. On the basis of the study objectives, it is hypothesized that children in the FHLP intervention group will show greater reductions in BMI *z* scores than those in the control group over the 12-month period. In addition, improvements in secondary outcomes are anticipated, including healthier lifestyle behaviors, enhanced quality of life for children, and increased parental self-efficacy and motivation to support their child’s healthy living. The blended delivery model, which combines self-guided resources with web-based synchronous sessions, is likely to foster greater engagement and adherence, promoting sustainable behavior changes.

### Comparison to Previous Work

The FHLP intervention was designed based on the M-PAC framework for supporting behavior change through reflective, regulatory, and reflexive processes. Several previous randomized controlled trials, including studies focusing on families with children in the target age range, have demonstrated that the M-PAC framework can be applied to promote a healthy lifestyle in community-based interventions [26,59-62].

Compared to existing family-based lifestyle interventions, the FHLP curriculum addresses a key gap by targeting 5 different behavioral domains to support healthy living (ie, physical activity, healthy eating, sleep, media use, and mental well-being). This has been identified as a strength because the majority of family-based childhood obesity interventions address only activity and nutrition [63].

Previous studies have found that eHealth behavior interventions can be effective for childhood obesity management, with technology-based delivery increasing intervention reach and accessibility [64,65]. However, participant engagement has been noted as a challenge for remote delivery [66]. This trial proposes engagement strategies that include weekly live sessions with a group facilitator, branded kit materials, frequent communication with participants, and personal check-ins from delivery staff to mitigate retention challenges.

This trial is one of the first to examine the long-term impact of blended web-based family-based behavioral interventions for childhood obesity. A recent randomized controlled trial of a 10-week web-based lifestyle intervention found it effective in improving short-term health outcomes for children with overweight or obesity, including decreasing BMI *z* scores and increasing quality of life, diet, and daily physical activity immediately after the intervention; however, long-term effectiveness has yet to be investigated [67].

### Strengths and Limitations

The study has several strengths. First, the innovative blended intervention design combines the scalability and flexibility of internet-based programs with the benefits of real-time support through web-based synchronous sessions. This hybrid approach has the potential to address common challenges in traditional in-person or self-guided web-based programs, such as limited accessibility and engagement, respectively. Second, the program was developed in collaboration with community stakeholders and informed by feedback from pilot participants, ensuring its relevance and practical applicability. Third, the intervention is grounded in the M-PAC framework, a robust theoretical model that emphasizes intention formation, action control, and maintenance, supporting sustained behavior change. Fourth, the rigorous trial design, which follows best practices for real-world randomized controlled trials [68,69], including pediatric-specific recommendations applied to reduce the risk of bias [70], enhances the validity and reliability of the findings. Finally, the study incorporates comprehensive outcome measures, providing a holistic understanding of the program’s impact on both children and parents.

However, this study also has limitations. The reliance on parent-reported home measurement data for height, weight, and dietary behaviors may introduce error due to variation between different scales. Protocols for parent-report measure for child height and weight were adopted due to COVID-19–related restrictions at the start of the trial. Previous research has shown that parent measurements of height and weight can be effective in classifying child weight status in the absence of professional measurements [71] and that providing instructional guides may improve accuracy [72]. Therefore, participating parents will be instructed to measure children as accurately as possible and provided with a link to a US Centers for Disease Control and Prevention guide titled “Measuring Children’s Height and Weight” [35].

Despite these limitations, this study has the potential to significantly advance the field of childhood obesity management by providing evidence on the effectiveness of an internet-based intervention. The results will offer valuable insights into how web-based synchronous sessions can enhance engagement, support behavior change, and improve health outcomes. These findings will inform policymakers, health care providers, and community organizations on the development and implementation of scalable, flexible, and effective interventions for managing childhood obesity.

### Dissemination Plan

This study is founded on the principles of integrated knowledge translation with an actively engaged academic, community, and public health partnership guiding the development of the intervention, the research and evaluation questions, the interpretation of results, and dissemination [73].

It is anticipated that the group-level deidentified results of this trial will be shared through conference presentations, reports to community stakeholders, and academic publications. A range of knowledge translation tools will also be developed for the general public, including dissemination of the resulting publications and general study information via web-based platforms, including the University of Victoria Digital Health Lab website, and official social media channels.

### Future Directions

The research team is already working closely with a community partner (the Childhood Healthy Living Foundation) and will continue to collaborate with knowledge users to ensure that research outcomes are responsive to shifting community and knowledge translation needs. Trial findings regarding the content and methodology of stand-alone and blended web-based interventions will be used to support evidence-informed policy decisions in future.

### Conclusions

The FHLP represents an innovative, scalable, and family-focused intervention designed to address the growing public health challenge of childhood obesity. Leveraging feedback from community stakeholders and pilot participants has allowed tailoring of the FHLP to meet the diverse needs of families while maintaining flexibility and accessibility. By integrating self-guided resources with web-based synchronous group sessions, the FHLP aims to enhance engagement, promote sustainable behavior changes, and improve BMI *z* scores and related health outcomes in children aged 8 to 12 years.

This trial will provide critical insights into the relative effectiveness of blended versus stand-alone web-based interventions, addressing a key gap in current literature. The findings from this study may inform future program development and implementation strategies, offering a practical, evidence-based solution to support families in adopting healthier lifestyles. Ultimately, the FHLP has the potential to make a meaningful contribution to public health efforts aimed at reducing childhood obesity and improving quality of life for children and their families.

## Appendix

Multimedia Appendix 1 Research consent form.

Multimedia Appendix 2 Peer review report from the Psychosocial, Sociocultural & Behavioural Determinants of Health Committee / Déterminants psychosociaux, socioculturels et comportementaux de la santé Comité - Canadian Institutes of Health Research (CIHR) / Institutsde recherche en santé du Canada (IRSC) (Canada).

## Abbreviations

CONSORT: Consolidated Standards of Reporting Trials
FHLP: Family Healthy Living Program
FLASHE: Family Life, Activity, Sun, Health, and Eating
M-PAC: multiprocess action control
REDCap: Research Electronic Data Capture
SPIRIT: Standard Protocol Items: Recommendations for Interventional Trials

## Data Availability

Anonymized group-level data as well as the statistical analysis plan and data dictionary will be available from the corresponding author on reasonable request.

## References

[1] Rao DP, Kropac E, Do MT, Roberts KC, Jayaraman GC (2016). Childhood overweight and obesity trends in Canada. Health Promot Chronic Dis Prev Can.

[2] Maximova K, Kuhle S, Davidson Z, Fung C, Veugelers PJ (2013). Cardiovascular risk-factor profiles of normal and overweight children and adolescents: insights from the Canadian Health Measures Survey. Can J Cardiol.

[3] Schwimmer JB, Burwinkle TM, Varni JW (2003). Health-related quality of life of severely obese children and adolescents. JAMA.

[4] Twig G, Yaniv G, Levine H, Leiba A, Goldberger N, Derazne E, Ben-Ami Shor D, Tzur D, Afek A, Shamiss A, Haklai Z, Kark JD (2016). Body-mass index in 2.3 million adolescents and cardiovascular death in adulthood. N Engl J Med.

[5] Sacks G, Shill J, Snowdon W, Swinburn B, Armstrong T, Irwin R, Randby S, Xuereb G (2012). Prioritizing areas for action in the field of population-based prevention of childhood obesity: a set of tools for Member States to determine and identify priority areas for action. World Health Organization.

[6] Richter LM, Daelmans B, Lombardi J, Heymann J, Boo FL, Behrman JR, Lu C, Lucas JE, Perez-Escamilla R, Dua T, Bhutta ZA, Stenberg K, Gertler P, Darmstadt GL (2017). Investing in the foundation of sustainable development: pathways to scale up for early childhood development. The Lancet.

[7] Allin S, Mossialos E, McKee M, Holland W (2004). Making decisions on public health: a review of eight countries. World Health Organization.

[8] Wang Y, Wu Y, Wilson RF, Bleich S, Cheskin L, Weston C, Showell N, Fawole O, Lau B, Segal J (2013). Childhood obesity prevention programs: comparative effectiveness review and meta-analysis. Agency for Healthcare Research and Quality.

[9] Liu S, Weismiller J, Strange K, Forster-Coull L, Bradbury J, Warshawski T, Naylor PJ (2020). Evaluation of the scale-up and implementation of mind, exercise, nutrition … do it! (MEND) in British Columbia: a hybrid trial type 3 evaluation. BMC Pediatr.

[10] Sung-Chan P, Sung YW, Zhao X, Brownson RC (2013). Family-based models for childhood-obesity intervention: a systematic review of randomized controlled trials. Obes Rev.

[11] Kalarchian MA, Levine MD, Arslanian SA, Ewing LJ, Houck PR, Cheng Y, Ringham RM, Sheets CA, Marcus MD (2009). Family-based treatment of severe pediatric obesity: randomized, controlled trial. Pediatrics.

[12] Croker H, Viner RM, Nicholls D, Haroun D, Chadwick P, Edwards C, Wells JC, Wardle J (2012). Family-based behavioural treatment of childhood obesity in a UK National Health Service setting: randomized controlled trial. Int J Obes (Lond).

[13] Savoye M, Shaw M, Dziura J, Tamborlane WV, Rose P, Guandalini C, Goldberg-Gell R, Burgert TS, Cali AM, Weiss R, Caprio S (2007). Effects of a weight management program on body composition and metabolic parameters in overweight children: a randomized controlled trial. JAMA.

[14] Hohman KH, Price SN, Sonneville K, Rifas-Shiman SL, Gortmaker SL, Gillman MW, Taveras EM (2012). Can the Internet be used to reach parents for family-based childhood obesity interventions?. Clin Pediatr (Phila).

[15] Antwi FA, Fazylova N, Garcon M, Lopez L, Rubiano R, Slyer JT (2013). Effectiveness of web-based programs on the reduction of childhood obesity in school-aged children: a systematic review. JBI Database System Rev Implement Rep.

[16] Delamater AM, Pulgaron ER, Rarback S, Hernandez J, Carrillo A, Christiansen S, Severson HH (2013). Web-based family intervention for overweight children: a pilot study. Child Obes.

[17] Liu S, Dunford SD, Leung YW, Brooks D, Thomas SG, Eysenbach G, Nolan RP (2013). Reducing blood pressure with internet-based interventions: a meta-analysis. Can J Cardiol.

[18] Mohr DC, Cuijpers P, Lehman K (2011). Supportive accountability: a model for providing human support to enhance adherence to eHealth interventions. J Med Internet Res.

[19] Pandita A, Sharma D, pandita D, Pawar S, kaul A, Tariq M (2016). Childhood obesity: prevention is better than cure. Diabetes Metab Syndr Obes.

[20] Curbing childhood obesity: a federal, provincial and territorial framework for action to promote healthy weights. Government of Canada.

[21] Liu S, Marques IG, Perdew MA, Strange K, Hartrick T, Weismiller J, Ball GD, Mâsse LC, Rhodes R, Naylor PJ (2019). Family-based, healthy living intervention for children with overweight and obesity and their families: a 'real world' trial protocol using a randomised wait list control design. BMJ Open.

[22] Rhodes RE, de Bruijn GJ (2013). What predicts intention-behavior discordance? A review of the action control framework. Exerc Sport Sci Rev.

[23] Rhodes RE, Yao CA (2015). Models accounting for intention-behavior discordance in the physical activity domain: a user's guide, content overview, and review of current evidence. Int J Behav Nutr Phys Act.

[24] Rhodes R, Grant S (2018). Bridging the intention-behavior gap in physical activity: a review of evidence from the multi-process action control framework. Ann Behav Med.

[25] Rhodes RE, Rebar AL (2017). Conceptualizing and defining the intention construct for future physical activity research. Exerc Sport Sci Rev.

[26] Kaushal N, Rhodes RE, Spence JC, Meldrum JT (2017). Increasing physical activity through principles of habit formation in new gym members: a randomized controlled trial. Ann Behav Med.

[27] Perdew M, Liu S, Rhodes R, Ball GD, Mâsse LC, Hartrick T, Strange K, Naylor PJ (2021). The effectiveness of a blended in-person and online family-based childhood obesity management program. Child Obes.

[28] Nuss K, Coulter R, DeSilva B, Buenafe J, Sheikhi R, Naylor PJ, Liu S (2022). Evaluating the effectiveness of a family-based virtual childhood obesity management program delivered during the COVID-19 pandemic in Canada: prospective study. JMIR Pediatr Parent.

[29] Moher D, Hopewell S, Schulz KF, Montori V, Gøtzsche PC, Devereaux PJ, Elbourne D, Egger M, Altman DG (2010). CONSORT 2010 explanation and elaboration: updated guidelines for reporting parallel group randomised trials. BMJ.

[30] Chan AW, Tetzlaff JM, Altman DG, Laupacis A, Gøtzsche PC, Krleža-Jerić K, Hróbjartsson A, Mann H, Dickersin K, Berlin JA, Doré CJ, Parulekar WR, Summerskill WS, Groves T, Schulz KF, Sox HC, Rockhold FW, Rennie D, Moher D (2013). SPIRIT 2013 statement: defining standard protocol items for clinical trials. Ann Intern Med.

[31] Harris PA, Taylor R, Thielke R, Payne J, Gonzalez N, Conde JG (2009). Research electronic data capture (REDCap)--a metadata-driven methodology and workflow process for providing translational research informatics support. J Biomed Inform.

[32] Harris PA, Taylor R, Minor BL, Elliott V, Fernandez M, O'Neal L, McLeod L, Delacqua G, Delacqua F, Kirby J, Duda SN (2019). The REDCap consortium: building an international community of software platform partners. J Biomed Inform.

[33] Sacher PM, Kolotourou M, Chadwick PM, Cole TJ, Lawson MS, Lucas A, Singhal A (2010). Randomized controlled trial of the MEND program: a family-based community intervention for childhood obesity. Obesity (Silver Spring).

[34] (2006). WHO child growth standards: length/height-for-age, weight-for-age, weight-for-length, weight-for-height and body mass index-for-age: methods and development. World Health Organization.

[35] (2024). Measuring children’s height and weight. Centers for Disease Control and Prevention.

[36] Longmuir PE, Gunnell KE, Barnes JD, Belanger K, Leduc G, Woodruff SJ, Tremblay MS (2018). Canadian Assessment of Physical Literacy Second Edition: a streamlined assessment of the capacity for physical activity among children 8 to 12 years of age. BMC Public Health.

[37] Kowalski KC, Crocker PR, Donen RM (2004). The physical activity questionnaire for older children (PAQ-C) and adolescents (PAQ-A) manual. Pediatric Research in Sports Medicine Society.

[38] Prochaska JJ, Sallis JF, Long B (2001). A physical activity screening measure for use with adolescents in primary care. Arch Pediatr Adolesc Med.

[39] Mâsse LC, Lytle LA (2017). Advancing knowledge of parent-child dyadic relationships about multiple cancer preventive health behaviors: the National Cancer Institute Family Life, Activity, Sun, Health, and Eating (FLASHE) study. Am J Prev Med.

[40] Silva NM (2014). The behavioral risk factor surveillance system. Int J Aging Hum Dev.

[41] Keihner AJ, Meigs R, Sugerman S, Backman D, Garbolino T, Mitchell P (2011). J Nutr Educ Behav.

[42] Wilson AM, Magarey AM, Mastersson N (2008). Reliability and relative validity of a child nutrition questionnaire to simultaneously assess dietary patterns associated with positive energy balance and food behaviours, attitudes, knowledge and environments associated with healthy eating. Int J Behav Nutr Phys Act.

[43] Lohse B, Cunningham-Sabo L, Walters LM, Stacey JE (2011). Valid and reliable measures of cognitive behaviors toward fruits and vegetables for children aged 9 to 11 years. J Nutr Educ Behav.

[44] French SA, Wall M, Corbeil T, Sherwood NE, Berge JM, Neumark-Sztainer D (2018). Obesity in adolescence predicts lower educational attainment and income in adulthood: the Project EAT Longitudinal Study. Obesity (Silver Spring).

[45] Froh JJ, Sefick WJ, Emmons RA (2008). Counting blessings in early adolescents: an experimental study of gratitude and subjective well-being. J Sch Psychol.

[46] Sutton E, Schonert-Reichl KA, Wu AD, Lawlor MS (2017). Evaluating the reliability and validity of the self-compassion scale short form adapted for children ages 8–12. Child Ind Res.

[47] Varni JW, Seid M, Kurtin PS (2001). PedsQL 4.0: reliability and validity of the Pediatric Quality of Life Inventory version 4.0 generic core scales in healthy and patient populations. Med Care.

[48] Rhodes RE, Blanchard CM, Matheson DH (2010). A multicomponent model of the theory of planned behaviour. British J Health Psychol.

[49] Wilson PM, Muon S (2008). Psychometric properties of the exercise identity scale in a university sample. Int J Sport Exerc Psychol.

[50] Lampard AM, Nishi A, Baskin ML, Carson TL, Davison KK (2016). The activity support scale for multiple groups (ACTS-MG): child-reported physical activity parenting in African American and non-Hispanic White families. Behav Med.

[51] Rhodes RE, Spence JC, Berry T, Deshpande S, Faulkner G, Latimer-Cheung AE, O'Reilly N, Tremblay MS (2016). Understanding action control of parental support behavior for child physical activity. Health Psychol.

[52] Nebeling LC, Hennessy E, Oh AY, Dwyer LA, Patrick H, Blanck HM, Perna FM, Ferrer RA, Yaroch AL (2017). The FLASHE study: survey development, dyadic perspectives, and participant characteristics. Am J Prev Med.

[53] Robinson-O'Brien R, Neumark-Sztainer D, Hannan PJ, Burgess-Champoux T, Haines J (2009). Fruits and vegetables at home: child and parent perceptions. J Nutr Educ Behav.

[54] Verplanken B, Orbell S (2006). Reflections on past behavior: a self-report index of habit strength. J Appl Soc Psychol.

[55] van de Kolk I, Verjans-Janssen SR, Gubbels JS, Kremers SP, Gerards SM (2019). Systematic review of interventions in the childcare setting with direct parental involvement: effectiveness on child weight status and energy balance-related behaviours. Int J Behav Nutr Phys Act.

[56] Sex and gender in health research. Canadian Institutes of Health Research.

[57] O'Reilly CJ (2018). A case study of the BalancedView course: addressing weight stigma among health care providers in British Columbia. University of British Columbia.

[58] CSEP Get Active Questionnaire. Canadian Society for Exercise Physiology.

[59] Zhou L, Liang W, He Y, Duan Y, Rhodes RE, Lippke S, Baker JS, Liang Y, Han L, Liu WX, Liu Q (2023). A school-family blended multi-component physical activity program for Fundamental Motor Skills Promotion Program for Obese Children (FMSPPOC): protocol for a cluster randomized controlled trial. BMC Public Health.

[60] Quinlan A, Rhodes RE, Blanchard CM, Naylor PJ, Warburton DE (2015). Family planning to promote physical activity: a randomized controlled trial protocol. BMC Public Health.

[61] Hollman H, Sui W, Rhodes RE (2022). A feasibility randomized controlled trial of a multi-process action control web-based intervention that targets physical activity in mothers. Women Health.

[62] Streight E, Beauchamp MR, Smith KJ, Blanchard CM, Carson V, Strachan SM, Vanderloo LM, Courtnall S, Rhodes RE (2024). "We are an active family": a randomized trial protocol to evaluate a family-system social identity intervention to promote child physical activity. BMC Public Health.

[63] Ash T, Agaronov A, Young T, Aftosmes-Tobio A, Davison KK (2017). Family-based childhood obesity prevention interventions: a systematic review and quantitative content analysis. Int J Behav Nutr Phys Act.

[64] Azevedo LB, Stephenson J, Ells L, Adu-Ntiamoah S, DeSmet A, Giles EL, Haste A, O'Malley C, Jones D, Chai LK, Burrows T, Collins CE, van Grieken A, Hudson M (2022). The effectiveness of e-health interventions for the treatment of overweight or obesity in children and adolescents: a systematic review and meta-analysis. Obes Rev.

[65] Laura L (2015). Technology as a platform for improving healthy behaviors and weight status in children and adolescents: a review. Obesity.

[66] Bradley LE, Smith-Mason CE, Corsica JA, Kelly MC, Hood MM (2019). Remotely delivered interventions for obesity treatment. Curr Obes Rep.

[67] Zhu D, Dordevic AL, Gibson S, Davidson ZE (2025). The effectiveness of a 10-week family-focused e-Health healthy lifestyle program for school-aged children with overweight or obesity: a randomised control trial. BMC Public Health.

[68] Zwarenstein M, Treweek S, Gagnier JJ, Altman DG, Tunis S, Haynes B, Oxman AD, Moher D (2008). Improving the reporting of pragmatic trials: an extension of the CONSORT statement. BMJ.

[69] Gluud LL (2006). Bias in clinical intervention research. Am J Epidemiol.

[70] DeHoff BA, Staten LK, Rodgers RC, Denne SC (2016). The role of online social support in supporting and educating parents of young children with special health care needs in the United States: a scoping review. J Med Internet Res.

[71] Ohri-Vachaspati P, Acciai F, DeLia D, Lloyd K, Yedidia MJ (2019). Accuracy of parent-measured and parent-estimated heights and weights in determining child weight status. JAMA Pediatr.

[72] Patel D, Vesely SK, Dev DA, Guseman EH, Hord N, Eliot K, Sisson SB (2024). Accuracy of parent-measured weight and height of preschool children at home with increasing levels of instruction. Child Obes.

[73] Graham ID, Tetroe J (2007). How to translate health research knowledge into effective healthcare action. Healthc Q.

