# Supplementary material for: Evaluating the Effectiveness of a Family-Based Lifestyle Intervention for Managing Childhood Overweight: Protocol for a Randomized Controlled Trial
Source: JMIR Res Protoc. 2025 Oct 14;14:e76837. doi: 10.2196/76837 (PMC12569496; doi:10.2196/76837)
Supplement: Multimedia Appendix 1 [file resprot_v14i1e76837_app1.pdf]

Research Consent Form

Generation Health Evaluation Study

You are invited to participate in the Generation Health Evaluation Study because you have expressed interest in participating in the Generation Health program and the evaluation is a core component of the program. If you join the program you are agreeing to participate in the evaluation component of the Generation Health study to evaluate whether the program is effective and feasible for families.

The Generation Health Evaluation is grant funded by the Canadian Institute of Health Research. The evaluation component is being carried out by Dr. Sam Liu, in collaboration with Dr. PJ Naylor, of the University of Victoria. You may contact them by phone or e-mail (Dr. Liu: 250-721-8392, samliu@uvic.ca; Dr. Naylor: 250-721-7844, pjnaylor@uvic.ca) if you have further questions.

Purpose and Objectives

The purpose of this study is to evaluate the efficacy of a stand-alone (Web Only) and Control group Generation Health program for improving healthy lifestyle behaviours.

Importance of this Study

Evaluation studies of this type are important because they help us understand how to help families adopt a healthy lifestyle that keeps them on a healthy lifestyle and healthy weight path.

What is involved?

Together, eligible children and their parent(s)/caregiver(s) will be randomly assigned to one of two groups: Web Only and Control. More than one child per family will be permitted to participate in the Generation Health program and evaluation if the child meets the specified inclusion criteria. Additionally, both parents are invited to participate in the program and evaluation, however only one parent is required to participate with their child(ren).

Web Only: Families in the Web Only group will participate in a 10-week web-based program with weekly online e-sessions each week. Sessions will cover topics such as: healthy eating, physical activity, behaviour change skills, positive mental health, parenting practices, and sleep hygiene. Participants will also engage in choice-based activities with family members each week. Sessions will cover topics such as: healthy eating, physical activity, behaviour change skills, positive mental health, parenting practices, and sleep hygiene.

Control: Families in the control group will receive a 10-week web-based program consisting of weekly educational email newsletters. The content of the control group will be similar to the Web-only format. However, the delivery format is different.

Parents/caregivers and children in both groups will be asked to complete measures on four separate occasions: baseline, 10 weeks, 6 months and 12 months. Children will be asked to complete questionnaires and physical measures (height and weight) and parents will be asked to complete questionnaires. Child physical activity levels will be tracked using an activity watch (e.g. FitBit). Data collected from the FitBit activity watch will include daily steps, exercise intensity (METS) and heart rate. FitBit data will not be streamed in real-time and location data will not be stored.

Please be advised that information about you that is gathered for this research study uses an app [FitBit] that stores data in the U.S. As such, there is a possibility that information about you may be accessed without your knowledge or consent by the US government in compliance with the US Freedom Act.

If you consent to voluntarily participate in the Generation Health evaluation, the research team will collect the information at the four time periods listed above. Your information will be combined with others for evaluation purposes. There will be no information in the research results that will be personally identifiable.

Inconvenience

Participation in this research may cause some additional inconvenience to you as we will be asking you to complete the measurements on your own time through your health care provider. Compensation for travel costs will hopefully mitigate this inconvenience. As well, to thank you for your time a $25 honorarium will be provided after the completion of each measurement at baseline, 10-week, 6-month and 12-month.

Risks

The potential risks of you and your child's participation in the Generation Health program evaluation include emotional distress due to the sensitive topics we are evaluating such as height and weight as well as lifestyle behaviors (i.e., physical activity, dietary behaviours). If you or your child experience distress during or after research participation and require assistance we recommend that you contact your general practitioner or call 8-1-1.

Benefits

By participating in this study, you are contributing to the evidence of Generation Health's ability to help children and families adopt a healthy lifestyle and stay on a healthy weight trajectory. Additionally, you are contributing to the evidence related to using online virtual programs for families working to improve their lifestyle behaviours and stay on a healthy weight path.

Voluntary Participation

Your participation in this program and evaluation study is completely voluntary. If you do decide to participate, you may withdraw at any time without any consequences or any explanation. If you do withdraw from the study you can choose to allow us to use your/your child's data collected to date or to not use it. If at any time you choose to withdraw consent, your preference for the use of your data will be documented and your request honoured.

Since participation in this study requires a child and caregiver to participate together, if you or your child/children decide to withdraw from the study, you will both have to withdraw; children cannot participate without a caregiver and caregivers cannot participate without a child.

On-going Consent

To make sure that you continue to consent to participate in the evaluation component, we will re-confirm your consent when at 10-week, 6-month and 12-month measurement sessions.

Anonymity

In terms of protecting your anonymity there will be no identifying names on any of your/your child's records. Your name/your child's name will be replaced by unique identification numbers. You will not be completely anonymous as the research team knows who is participating.

Confidentiality

Your confidentiality and the confidentiality of the data will be protected by having no participant names on any of the data. As well, hard copies of the data will be stored in a locked filing cabinet in a locked room at the University of Victoria. Electronic files will be stored using your unique identification numbers on a secure network drive at the University of Victoria which is accessible only to the principal investigator and research staff.

Access to Data

Access to your child's health information (e.g. height, weight, questionnaire responses etc.) collected at the measurement sessions will be available upon request.

Dissemination of Results

It is anticipated that the results of this evaluation study will be shared with others through presentations at conferences, a report to public health and community stakeholders, academic publications and thesis/dissertation/class presentations.

Disposal of Data

This data (e.g. questionnaires, height and weight, physical activity levels) will be disposed of five years following publication. If the results are not published within 5 years of completing the study, the data will be destroyed. Hard copies will be shredded and any computer files with participant information will be deleted.

Individuals that may be contacted regarding this study include:

Dr. Sam Liu	Dr. PJ Naylor
Principal investigator	Co-investigator
250-721-8392	250-721-7844
samliu@uvic.ca	pjnaylor@uvic.ca

In addition, you may verify the ethical approval of this study, or raise any concerns you might have, by contacting the Human Research Ethics Office at the University of Victoria (250-472-4545 or ethics@uvic.ca).

Your electronic signature below indicates that you understand the above conditions of participation in this study, that you have had the opportunity to have your questions answered by the researchers, and that you consent to participate in this study.

Please provide your first and last name

____

Please provide your email address

____

How many children do you have registered for the Generation Health program?

(As a reminder, only children between the ages of 8 and 12 are officially registered. However, we do hope that your whole family will participate in the program sessions and the family-based challenges! All siblings are welcome to attend the Zoom sessions and join the activities.)

- ◯ One child
- ◯ Two children
- ◯ Three children

Children's Statement:

I have talked with my parents/guardians about the Generation Health program and evaluation study and I understand that all activities are a normal part of the Generation Health Program. I understand that if I want to, I can stop being in the program evaluation study at any time. I have had the chance to ask questions and have received satisfactory answers to all of my questions.

Name of child one

____

Name of child two

____

Name of child three ______________________

We ask for each child's height and weight to track their growth trajectory - please try to measure as carefully as possible! Accurate measurements will be very important. To learn how to measure your child's height and weight accurately, click here.

What is [name_child1]'s measured height in centimeters? ______________________

What is [name_child1]'s measured weight in pounds? ______________________

What is [name_child2]'s measured height in centimeters? ______________________

What is [name_child2]'s measured weight in pounds? ______________________

What is [name_child3]'s measured height in centimeters? ______________________

What is [name_child3]'s measured weight in pounds? ______________________

Today's date ______________________
