# Supplementary material for: Evaluating the Effectiveness of a Family-Based Lifestyle Intervention for Managing Childhood Overweight: Protocol for a Randomized Controlled Trial
Source: JMIR Res Protoc. 2025 Oct 14;14:e76837. doi: 10.2196/76837 (PMC12569496; doi:10.2196/76837)
Supplement: Multimedia Appendix 2 [file resprot_v14i1e76837_app2.pdf]

**Review Type / Type d'évaluation:**	Reviewer 1 / Évaluateur 1
**Name of Applicant / Nom du chercheur:**	Liu, Sam
**Application No. / Numéro de demande:**	427406
**Agency / Agence:**	CIHR/IRSC
**Competition / Concours:**	Project Grant/Subvention Projet
**Committee / Comité:**	Psychosocial, Sociocultural & Behavioural Determinants of Health/Déterminants psychosociaux, socioculturels et comportementaux de la santé
**Title / Titre:**	Childhood Obesity Management Using Innovative Digital Technology: A "Real World" Randomized Community Trial

Adjudication Criteria/Critères de sélection

Significance and Impact of the Research/Importance et impact de la recherche: 4.2

Approaches and Methods/Approches et méthodes: 3.9

Expertise, Experience and Resources/Expertise, expérience et ressources: 4.3

Top/Bottom Selection/Groupe supérieur/inférieur

- (●) **Top/Groupe supérieur**
- ( ) **Bottom/Groupe inférieur**

**Review Type / Type d'évaluation:**	Reviewer 1 / Évaluateur 1
**Name of Applicant / Nom du chercheur:**	Liu, Sam
**Application No. / Numéro de demande:**	427406
**Agency / Agence:**	CIHR/IRSC
**Competition / Concours:**	Project Grant/Subvention Projet
**Committee / Comité:**	Psychosocial, Sociocultural & Behavioural Determinants of Health/Déterminants psychosociaux, socioculturels et comportementaux de la santé
**Title / Titre:**	Childhood Obesity Management Using Innovative Digital Technology: A "Real World" Randomized Community Trial

Summary of Application/Résumé de la demande:

The proposed study is a Randomized Controlled Trail (RCT) designed to determine the efficacy of delivering a stand alone web-based Family Health Living Program (FHLP) and a blended in person and web-based FHLP, relative to a control group in children 8-12 years with elevated BMI for their age and sex (>=85th percentile), on zBMI at 4- and 12-month follow-ups. Secondary outcomes include: lifestyle behaviors, health related quality of life, parent self-efficacy and motivation to support their families in healthy living.

Embedded within the study is also an economic evaluation that will include a description of the costs associated with delivering each intervention and a cost-effectiveness analysis of the Web Only and Blended FHLP relative to a control group.

The blended FHLP includes weekly, 2-hour in person healthy living group sessions for 4 months (16 sessions). The sessions will be led by a trained facilitator. The first hour includes learning about healthy living skills and the 2nd hour is interactive. The sessions include parent-only, child-only and family components. Facilitators try to emphasize behaviour change through cognitive and behavioral techniques (such as goal setting and monitoring). Families will also have access to an online family portal that offers additional resources (e.g., healthy recipes, articles for parents, suggested healthy eating and physical activities). It will be a repository of materials covered during each session, so participants can access intervention content outside of the face-to-face sessions

The Web only FHLP includes the same content and scheduled weekly lessons as the Blended FHLP group. They can access the content via a secure, web portal. The web only is designed for parents, although it has child-specific interactive activities. The online sessions will be tailored using smart algorithms to factor in: culture and background, priorities and motivation for change in family behaviors, and objective measures of weekly lifestyle behaviors. families will have access to a moderated secure online community where they can communicate with the facilitators and other families.

The control group will receive weekly educational newsletters consisting of information from the Canadian Public Health Agency (guides to healthy living, food guide, physical activity).

The proposal is a re-submission and the applicants have addressed many of the previous reviewers’ comments.

Strengths and Weaknesses/Forces et faiblesses:

Strengths

1.The applicants have established strong research collaborations with key stakeholder groups, including the BC Ministry of Health and the Childhood Obesity Foundation, helping to ensure the feasibility, applicability and scalability of the interventions being trialed.

2.The use of technology to deliver FHLP interventions has the potential to address challenges of access, reach and scalability, features that are incredibly important for public health interventions.

3.The inclusion of 6 sites across BC, both urban and rural, is another strength of the study design, in seeking to address barriers to access amongst families living in more remote areas.

4.The applicants developed the Web-Only and Blended FHLP interventions in partnership with the BC Ministry of Health and Childhood Obesity and consulted with a large number of provincial stakeholders cutting across multiple sectors (health, physical activity, recreation and education).

5.The applicants also completed a pilot feasibility study with both interventions across 6 urban and rural sites in BC. The blended FHLP included 54 children and demonstrated moderate effect sizes on the primary outcome (z-BMI; d=0.3) and secondary outcomes.

6.The web-only FHLP uses smart algorithms to personalize weekly lessons and incorporates behavior techniques (performance feedback, self-monitoring, modeling), proactive reminder notifications, and gaming techniques to improve engagement

7.The team is interdisciplinary in nature covering off expertise across the various aspects of the grant, including eHealth and health promotion, pediatric obesity, behavioral science, biostatistics, and cost-effectiveness evaluation. They are well-positioned to successfully implement the RCT and disseminate their findings to academic audiences and key stakeholder groups and knowledge users, given their strong and established partnerships.

8.Strong integrated Knowledge translation plan that includes academics, community and public health partnerships guiding the development of the intervention, the research questions, interpretation of the results and dissemination

Weaknesses

1.The approach to screening for participant eligibility is not described. Will interested families call the study office and the RC will assess eligibility? How will BMI be determined? Is it based on parent self-report over the phone? The applicants also note that participants with diagnoses of psychological/behavioral issues will not be eligible to participate, but a rationale for this decision is not provided, nor do they document how this determination will be made. There is strong evidence documenting a robust association between BMI and mental health concerns, and excluding children on the basis of these concerns seems counterintuitive in an efficacy trial.

2.The description and proposed flexibility of the Web-Only FHLP is somewhat unclear, with respect to timing –are participants expected to engage in the weekly sessions at a set/pre-specified time or do they have the flexibility to engage in these lessons at any time that is convenient to them? Will there be an on-line facilitator engaged in the weekly lessons for the Web-Only FHLP?

3.Does the Blended FHLP get any of the innovative smart technological benefits (i.e., tailored messaging, reminders,

performance feedback) or is the web-portion of this intervention simply the web-portal with resources?

4.The sample determination does not specify the anticipated effect sizes for each of the interventions, relative to the control group.
5.The data analyses section specifies that the analyses will adjust for dependency between dyads (parent relationship to child) but the rationale for this co-variate control is unclear.
6.The collection of physical activity data using accelerometery is now well described. Will children wear the Actigraph for the full duration of the study? And how will the amount of data be managed and analyzed?
7.An important consideration are possible site differences that have not been acknowledged, particularly given that the sites differ along the urban-rural continuum. How will clustering of participants within sites be addressed? Participants within sites may be more similar compared to participants across sites. If within site correlations exist, then multiple regression approaches are not warranted given the inability to meet the underlying assumption of independence (i.e., potentially contributing to biased/under-estimates of SEs and inflated p-values).

**Review Type / Type d'évaluation:**	Reviewer 1 / Évaluateur 1
**Name of Applicant / Nom du chercheur:**	Liu, Sam
**Application No. / Numéro de demande:**	427406
**Agency / Agence:**	CIHR/IRSC
**Competition / Concours:**	Project Grant/Subvention Projet
**Committee / Comité:**	Psychosocial, Sociocultural & Behavioural Determinants of Health/Déterminants psychosociaux, socioculturels et comportementaux de la santé
**Title / Titre:**	Childhood Obesity Management Using Innovative Digital Technology: A "Real World" Randomized Community Trial

Budget Recommendation/Recommandation budgétaire:

Please indicate your appraisal of the integration of sex as a biological variable as a strength, weakness, or not applicable to the proposal./Prière de sélectionner une option pour donner votre évaluation de l'intégration du sexe comme variable biologique en tant que point fort ou point faible de la proposition, ou en tant qu'élément non applicable à la proposition.

- (●) **Strength/Point fort**
- ( ) **Weakness/Point faible**
- ( ) **Not applicable/Non applicable**

Please indicate your appraisal of the integration of gender as a socio-cultural determinant of health as a strength, weakness, or not applicable to the proposal./Prière de sélectionner une option pour donner votre évaluation de l'intégration du genre comme déterminant socioculturel de la santé en tant que point fort ou point faible de la proposition, ou en tant qu'élément non applicable à la proposition.

- (●) **Strength/Point fort**
- ( ) **Weakness/Point faible**
- ( ) **Not applicable/Non applicable**

**Review Type / Type d'évaluation:**	Reviewer 1 / Évaluateur 1
**Name of Applicant / Nom du chercheur:**	Liu, Sam
**Application No. / Numéro de demande:**	427406
**Agency / Agence:**	CIHR/IRSC
**Competition / Concours:**	Project Grant/Subvention Projet
**Committee / Comité:**	Psychosocial, Sociocultural & Behavioural Determinants of Health/Déterminants psychosociaux, socioculturels et comportementaux de la santé
**Title / Titre:**	Childhood Obesity Management Using Innovative Digital Technology: A "Real World" Randomized Community Trial

Sex and/or Gender Considerations/Notions de sexe et/ou de genre:

The applicants will take gender and sex into account, by adjusting for gender in their analyses and examining potential sub-group differences by both sex and gender. Parents will complete the Gender Identity Questionnaire for Children.

**Review Type / Type d'évaluation:**	Reviewer 2 / Évaluateur 2
**Name of Applicant / Nom du chercheur:**	Liu, Sam
**Application No. / Numéro de demande:**	427406
**Agency / Agence:**	CIHR/IRSC
**Competition / Concours:**	Project Grant/Subvention Projet
**Committee / Comité:**	Psychosocial, Sociocultural & Behavioural Determinants of Health/Déterminants psychosociaux, socioculturels et comportementaux de la santé
**Title / Titre:**	Childhood Obesity Management Using Innovative Digital Technology: A "Real World" Randomized Community Trial

Adjudication Criteria/Critères de sélection

Significance and Impact of the Research/Importance et impact de la recherche: 4.6

Approaches and Methods/Approches et méthodes: 4.3

Expertise, Experience and Resources/Expertise, expérience et ressources: 4.2

Top/Bottom Selection/Groupe supérieur/inférieur

◉ **Top/Groupe supérieur**
○ **Bottom/Groupe inférieur**

**Review Type / Type d'évaluation:**	Reviewer 2 / Évaluateur 2
**Name of Applicant / Nom du chercheur:**	Liu, Sam
**Application No. / Numéro de demande:**	427406
**Agency / Agence:**	CIHR/IRSC
**Competition / Concours:**	Project Grant/Subvention Projet
**Committee / Comité:**	Psychosocial, Sociocultural & Behavioural Determinants of Health/Déterminants psychosociaux, socioculturels et comportementaux de la santé
**Title / Titre:**	Childhood Obesity Management Using Innovative Digital Technology: A "Real World" Randomized Community Trial

Summary of Application/Résumé de la demande:

This is a revised application proposing a randomized clinical trial comparing the efficacy and cost-effectiveness of a family-focused behavioural weight management program for children aged 8 to 12 years old delivered via a web-based platform or a blended approach combining web-based with a face-to-face intervention.

**Review Type / Type d'évaluation:**	Reviewer 2 / Évaluateur 2
**Name of Applicant / Nom du chercheur:**	Liu, Sam
**Application No. / Numéro de demande:**	427406
**Agency / Agence:**	CIHR/IRSC
**Competition / Concours:**	Project Grant/Subvention Projet
**Committee / Comité:**	Psychosocial, Sociocultural & Behavioural Determinants of Health/Déterminants psychosociaux, socioculturels et comportementaux de la santé
**Title / Titre:**	Childhood Obesity Management Using Innovative Digital Technology: A "Real World" Randomized Community Trial

Strengths and Weaknesses/Forces et faiblesses:

The application retains all of its original strengths including its focus on a major public health problem, its innovative use of digital technology to deliver an evidence-based intervention, extensive pilot data and an excellent knowledge transfer plan. In addition, this proposal is a natural extension of the applicant's previous work and there is a solid network of collaborators from both the academic and community settings.

Despite the many positive features of the original proposal, some methodological issues were raised as likely to compromise the interpretation of the findings and to limit the potential impact of the proposed study. Most of the issues about selection criteria, the outcome measures, and treatment fidelity check have been addressed adequately and the revised proposal is much stronger. However, a few relatively minor issues would still require further consideration.

The plan is to recruit at six different sites to increase the study feasibility. While recruitment sites appear to be diversified in terms of rural and urban areas, it might be best for the investigators to make sure that there is an adequate representation in the final sample of the different groups particularly at-risk for obesity (e.g., lower education and socioeconomic status, indigenous). Often times, these groups are more difficult to recruit and engage in such clinical trial and the final sample is not always representative of these subgroups. It would be best if the targeted sample were stratified to ensure adequate representation of known groups at risk for obesity.

In this revised proposal, the investigator addresses the expected attrition. Surprisingly, however, attrition is expected to be comparable across all conditions. Aside from some pilot studies, there is no strong basis for such expectations. Perhaps it would be important to examine not only subjects loss to follow up (true drop outs) but also examine treatment adherence and engagement as these might yield quite different data across an intervention that is entirely self-driven compared to one that includes complementary face-to-face support.

**Review Type / Type d'évaluation:**	Reviewer 2 / Évaluateur 2
**Name of Applicant / Nom du chercheur:**	Liu, Sam
**Application No. / Numéro de demande:**	427406
**Agency / Agence:**	CIHR/IRSC
**Competition / Concours:**	Project Grant/Subvention Projet
**Committee / Comité:**	Psychosocial, Sociocultural & Behavioural Determinants of Health/Déterminants psychosociaux, socioculturels et comportementaux de la santé
**Title / Titre:**	Childhood Obesity Management Using Innovative Digital Technology: A "Real World" Randomized Community Trial

Budget Recommendation/Recommandation budgétaire:

Appropriate as requested

**Review Type / Type d'évaluation:**	Reviewer 2 / Évaluateur 2
**Name of Applicant / Nom du chercheur:**	Liu, Sam
**Application No. / Numéro de demande:**	427406
**Agency / Agence:**	CIHR/IRSC
**Competition / Concours:**	Project Grant/Subvention Projet
**Committee / Comité:**	Psychosocial, Sociocultural & Behavioural Determinants of Health/Déterminants psychosociaux, socioculturels et comportementaux de la santé
**Title / Titre:**	Childhood Obesity Management Using Innovative Digital Technology: A "Real World" Randomized Community Trial

Please indicate your appraisal of the integration of sex as a biological variable as a strength, weakness, or not applicable to the proposal./Prière de sélectionner une option pour donner votre évaluation de l'intégration du sexe comme variable biologique en tant que point fort ou point faible de la proposition, ou en tant qu'élément non applicable à la proposition.

- (●) **Strength/Point fort**
- ( ) **Weakness/Point faible**
- ( ) **Not applicable/Non applicable**

Please indicate your appraisal of the integration of gender as a socio-cultural determinant of health as a strength, weakness, or not applicable to the proposal./Prière de sélectionner une option pour donner votre évaluation de l'intégration du genre comme déterminant socioculturel de la santé en tant que point fort ou point faible de la proposition, ou en tant qu'élément non applicable à la proposition.

- (●) **Strength/Point fort**
- ( ) **Weakness/Point faible**
- ( ) **Not applicable/Non applicable**

**Review Type / Type d'évaluation:**	Reviewer 2 / Évaluateur 2
**Name of Applicant / Nom du chercheur:**	Liu, Sam
**Application No. / Numéro de demande:**	427406
**Agency / Agence:**	CIHR/IRSC
**Competition / Concours:**	Project Grant/Subvention Projet
**Committee / Comité:**	Psychosocial, Sociocultural & Behavioural Determinants of Health/Déterminants psychosociaux, socioculturels et comportementaux de la santé
**Title / Titre:**	Childhood Obesity Management Using Innovative Digital Technology: A "Real World" Randomized Community Trial

Sex and/or Gender Considerations/Notions de sexe et/ou de genre:

**Review Type / Type d'évaluation:**	Reviewer 3 / Évaluateur 3
**Name of Applicant / Nom du chercheur:**	Liu, Sam
**Application No. / Numéro de demande:**	427406
**Agency / Agence:**	CIHR/IRSC
**Competition / Concours:**	Project Grant/Subvention Projet
**Committee / Comité:**	Psychosocial, Sociocultural & Behavioural Determinants of Health/Déterminants psychosociaux, socioculturels et comportementaux de la santé
**Title / Titre:**	Childhood Obesity Management Using Innovative Digital Technology: A "Real World" Randomized Community Trial

Adjudication Criteria/Critères de sélection

Significance and Impact of the Research/Importance et impact de la recherche: 4.1

Approaches and Methods/Approches et méthodes: 3.9

Expertise, Experience and Resources/Expertise, expérience et ressources: 3.7

Top/Bottom Selection/Groupe supérieur/inférieur

- (●) **Top/Groupe supérieur**
- ( ) **Bottom/Groupe inférieur**

**Review Type / Type d'évaluation:**	Reviewer 3 / Évaluateur 3
**Name of Applicant / Nom du chercheur:**	Liu, Sam
**Application No. / Numéro de demande:**	427406
**Agency / Agence:**	CIHR/IRSC
**Competition / Concours:**	Project Grant/Subvention Projet
**Committee / Comité:**	Psychosocial, Sociocultural & Behavioural Determinants of Health/Déterminants psychosociaux, socioculturels et comportementaux de la santé
**Title / Titre:**	Childhood Obesity Management Using Innovative Digital Technology: A "Real World" Randomized Community Trial

Summary of Application/Résumé de la demande:

Family-based interventions for obesity management have traditionally relied on face-to-face delivery, which limits the reach and scalability of the model. With increasing feasibility of web-based intervention delivery, there is a need to examine the efficacy and cost-effectiveness of these complementary approaches. The purpose of this study is to therefore evaluate the efficacy of a stand-alone (Web Only) and a blended in-person and web-based (Blended) Family Healthy Living Program (FHLP) relative to a control group for: i) improving body mass index (BMI z-scores), lifestyle behaviors, and quality of life in children (8–12 years) with elevated BMI; ii) to examine parental self-efficacy and motivation to support their families, and; iii) to conduct an economic evaluation to capture intervention delivery costs and subsequent cost-effectiveness. Using a a randomized controlled trial, n=342 parent-child dyads from six communities in British Columbia will be randomized and compared at baseline, 4, and 12 month follow-ups.

**Review Type / Type d'évaluation:**	Reviewer 3 / Évaluateur 3
**Name of Applicant / Nom du chercheur:**	Liu, Sam
**Application No. / Numéro de demande:**	427406
**Agency / Agence:**	CIHR/IRSC
**Competition / Concours:**	Project Grant/Subvention Projet
**Committee / Comité:**	Psychosocial, Sociocultural & Behavioural Determinants of Health/Déterminants psychosociaux, socioculturels et comportementaux de la santé
**Title / Titre:**	Childhood Obesity Management Using Innovative Digital Technology: A "Real World" Randomized Community Trial

Strengths and Weaknesses/Forces et faiblesses:

Research Team: NPA has an emerging record of HQP supervision, funding and publications (n=19), with 9 publications since first appointment in 2017. The remainder of research team is strong, has an established record of collaboration (iKT) with project partners, and has the skill set and experience to complete the proposed work. Use of a third party recruitment company will help to ensure recruitment targets are met; when taken together, the feasibility of the project is quite high.

Tailored intervention program using smart algorithm is well developed, and has some novel aspects such as culturally adapted / sensitive program information, a focus on sleep hygiene, etc. Feasibility of the approach has been demonstrated in pilot work.

Response to Reviewers: Previous reviewer comments were largely addressed by including a project timeline in the appendix and elaborating on the plan for HQP.

Limitations

While the analysis plan is generally well described, several elements of the statistical approach warrant clarification. Specifically, details of the exploratory (mediation and moderation) analyses -which will be used to better understand who the intervention works best for - warrant expansion, and should include detail on what dose-response questions will be explored (i.e. total activity by actigraphy, BMI, etc.). It is also not clear how nesting of 6 centers is to be accounted for and should be made explicit.

**Review Type / Type d'évaluation:**	Reviewer 3 / Évaluateur 3
**Name of Applicant / Nom du chercheur:**	Liu, Sam
**Application No. / Numéro de demande:**	427406
**Agency / Agence:**	CIHR/IRSC
**Competition / Concours:**	Project Grant/Subvention Projet
**Committee / Comité:**	Psychosocial, Sociocultural & Behavioural Determinants of Health/Déterminants psychosociaux, socioculturels et comportementaux de la santé
**Title / Titre:**	Childhood Obesity Management Using Innovative Digital Technology: A "Real World" Randomized Community Trial

Budget Recommendation/Recommandation budgétaire:

No concerns to note.

**Review Type / Type d'évaluation:**	Reviewer 3 / Évaluateur 3
**Name of Applicant / Nom du chercheur:**	Liu, Sam
**Application No. / Numéro de demande:**	427406
**Agency / Agence:**	CIHR/IRSC
**Competition / Concours:**	Project Grant/Subvention Projet
**Committee / Comité:**	Psychosocial, Sociocultural & Behavioural Determinants of Health/Déterminants psychosociaux, socioculturels et comportementaux de la santé
**Title / Titre:**	Childhood Obesity Management Using Innovative Digital Technology: A "Real World" Randomized Community Trial

Please indicate your appraisal of the integration of sex as a biological variable as a strength, weakness, or not applicable to the proposal./Prière de sélectionner une option pour donner votre évaluation de l'intégration du sexe comme variable biologique en tant que point fort ou point faible de la proposition, ou en tant qu'élément non applicable à la proposition.

- (●) **Strength/Point fort**
- ( ) **Weakness/Point faible**
- ( ) **Not applicable/Non applicable**

Please indicate your appraisal of the integration of gender as a socio-cultural determinant of health as a strength, weakness, or not applicable to the proposal./Prière de sélectionner une option pour donner votre évaluation de l'intégration du genre comme déterminant socioculturel de la santé en tant que point fort ou point faible de la proposition, ou en tant qu'élément non applicable à la proposition.

- (●) **Strength/Point fort**
- ( ) **Weakness/Point faible**
- ( ) **Not applicable/Non applicable**

**Review Type / Type d'évaluation:**	Reviewer 3 / Évaluateur 3
**Name of Applicant / Nom du chercheur:**	Liu, Sam
**Application No. / Numéro de demande:**	427406
**Agency / Agence:**	CIHR/IRSC
**Competition / Concours:**	Project Grant/Subvention Projet
**Committee / Comité:**	Psychosocial, Sociocultural & Behavioural Determinants of Health/Déterminants psychosociaux, socioculturels et comportementaux de la santé
**Title / Titre:**	Childhood Obesity Management Using Innovative Digital Technology: A "Real World" Randomized Community Trial

Sex and/or Gender Considerations/Notions de sexe et/ou de genre:

SGBA proposes to determine if treatment efficacy is different when gender vs sex is used; the overall question is described in the GSBA overview (in direct response to previous reviewer comments), and the analytic approach (regression) will examine gender.

Review Type/Type d'évaluation: SO Notes /Notes de l'agent scientifique
Name of Applicant/Nom du chercheur: Liu, Sam Xiaozhou
Application No./Numéro de demande: 427406
Agency/Agence: CIHR/IRSC
Competition/Concours: 2019-09-11 Project Grant/Subvention Projet
Committee/Comité: Psychosocial, Sociocultural & Behavioural Determinants of Health/Déterminants psychosociaux, socioculturels et comportementaux de la santé
Title/Titre: Childhood Obesity Management Using Innovative Digital Technology: A "Real World" Randomized Community Trial

---

Assessment/Évaluation:

Strengths:

This RCT resubmission was well received by the committee. The focus on increasing reach and accessibility was viewed as a strength, as was the economic evaluation and the inclusion of rural and urban communities. There are strong collaborations with key stakeholder groups, the use of technology is innovative, extensive pilot data were collected and the KT plan is well integrated to the design.

Weaknesses:

The committee discussed some minor issues. Participant eligibility screening, specifically with behavioral issues and BMI, would require more justification. A discussion of potential site differences would have strengthened this application. Effect sizes for determining sample size were not included. Representativeness of the sample was questioned and may need more attention. Comparing one intervention to the other in the cost analysis would improve the already strong design of this study. Adherence to and engagement with the online intervention should be measured. Additional clinical trial expertise would benefit the team. Overall the committee felt that this is an important study.

Budget: No issue.
Term: No issue
Eligibility: No issue
Ethics: No issue
